# Repositioning of Thiourea-Containing Drugs as Tyrosinase Inhibitors

**DOI:** 10.3390/ijms161226114

**Published:** 2015-12-02

**Authors:** Joonhyeok Choi, Jun-Goo Jee

**Affiliations:** Research Institute of Pharmaceutical Sciences, College of Pharmacy, Kyungpook National University, 80 Daehak-ro, Buk-gu, Daegu 702-701, Korea; crowz124@naver.com

**Keywords:** cheminformatics, docking simulation, drug repositioning, thiourea, tyrosinase

## Abstract

Tyrosinase catalyzes two distinct sequential reactions in melanin biosynthesis: The hydroxylation of tyrosine to dihydroxyphenylalanine (DOPA) and the oxidation of DOPA to dopaquinone. Developing functional modulators of tyrosinase is important for therapeutic and cosmetic purposes. Given the abundance of thiourea moiety in known tyrosinase inhibitors, we studied other thiourea-containing drugs as potential tyrosinase inhibitors. The thiourea-containing drugs in clinical use were retrieved and tested for their ability to inhibit tyrosinase. We observed that methimazole, thiouracil, methylthiouracil, propylthiouracil, ambazone, and thioacetazone inhibited mushroom tyrosinase. Except for methimazole, there was limited information regarding the activity of other drugs against tyrosinase. Both thioacetazone and ambazone significantly inhibited tyrosinase, with IC_50_ of 14 and 15 μM, respectively. Ambazone decreased melanin content without causing cellular toxicity at 20 μM in B16F10 cells. The activity of ambazone was stronger than that of kojic acid both in enzyme and melanin content assays. Kinetics of enzyme inhibition assigned the thiourea-containg drugs as non-competitive inhibitors. The complex models by docking simulation suggested that the intermolecular hydrogen bond via the nitrogen of thiourea and the contacts via thione were equally important for interacting with tyrosinase. These data were consistent with the results of enzyme assays with the analogues of thiourea.

## 1. Introduction

Type-3 copper proteins, including tyrosinase, catechol oxidase, and hemocyanin, possess six evolutionarily-conserved histidines. One copper ion chelates three histidines, and two adjacent copper ions form a catalytic center. Hemocyanin carries oxygen, whereas tyrosinase and catechol oxidase oxidize polyphenol. Tyrosinase facilitates the hydroxylation of monophenol, which catechol oxidase cannot catalyze. Tyrosinase, therefore, induces two distinct sequential reactions in living species, the hydroxylation of tyrosine to dihydroxyphenylalanine (DOPA) and the oxidation of DOPA to dopaquinone, leading to spontaneous production of melanin. Catalytic activities of tyrosinase are coupled with four possible oxidation states of copper ions: oxy-, met-, deoxy-, and deact-states [1]. Two Cu(II) ions form a planar structure with two oxygen atoms in oxy-states, whereas met- and deoxy-forms exist as [Cu(II)–Cu(II)] and [Cu(I)–Cu(I)], respectively. Two copper atoms are bridged by one or two hydroxide molecules in met-states. The coordinates around copper are disrupted in deact-states.

Melanin content plays critical roles in determining skin, eye, and hair color, as well as the browning of food. Congenital absence or defect of tyrosinase causes albinism, a disorder of melanin production in the body. Excess melanin due to overactive tyrosinase is also linked to skin disorders. Other diseases, including cancer and Parkinson’s disease, are characterized by abnormalities in tyrosinase activity [2,3,4,5]. Therefore, numerous natural and synthetic compounds that modulate the activity of tyrosinase have been discovered [6,7,8,9,10,11,12,13]. Inhibitors from natural resources include polyphenolic compounds, such as arbutin, which has been used as a skin-lightening agent. Phenylthiourea (PTU) and its synthetic derivatives comprise another well-known major class of tyrosinase inhibitors.

Considering the number of inhibitors, reports of the complex structures between tyrosinase and its inhibitors are limited. A couple of complex structures, one between PTU and sweet potato catechol oxidase (PDB ID: 1BUG) [14] and the other between tropolone and mushroom tyrosinase (PDB ID: 2Y9X) [15], have been published. Interestingly, these structures revealed that the inhibitors bind to the proteins in met and not in oxy-states. The recent crystal structures of substrates, tyrosine and DOPA, and tyrosinase from *Bacillus megaterium* captured intermediate structures in the enzyme reaction in states where copper ions were replaced with zinc atoms [16]. The structures provided a snapshot of the subtle changes in orientation for the oxidations of monophenol and catechol, uncovering common and distinct features of tyrosinase and catechol oxidase. The complex structure between catechol oxidase and PTU suggested that the thiourea or thiocarbamide moiety of PTU is crucial for recognizing proteins through the direct interaction with the catalytic copper ions.

Drug repositioning is the process of identifying new uses for existing drugs or drugs being tested in late clinical stages [17]. This approach is believed to reduce the risks associated with the traditional drug discovery process, particularly time and cost. We have reported that the analogues of ethionamide, a drug used for multidrug-resistant tuberculosis, share remote similarity with PTU and exhibit potent inhibition of tyrosinase [18]. In this study, we retrieved the drugs in clinical use that possess thiourea and investigated their effect on tyrosinase activities by using enzyme- and cell-based assays. The simulated binding modes between protein and small molecules are discussed.

## 2. Results and Discussion

### 2.1. Thoiurea Itself Inhibits Tyrosinase

**Figure 1:**
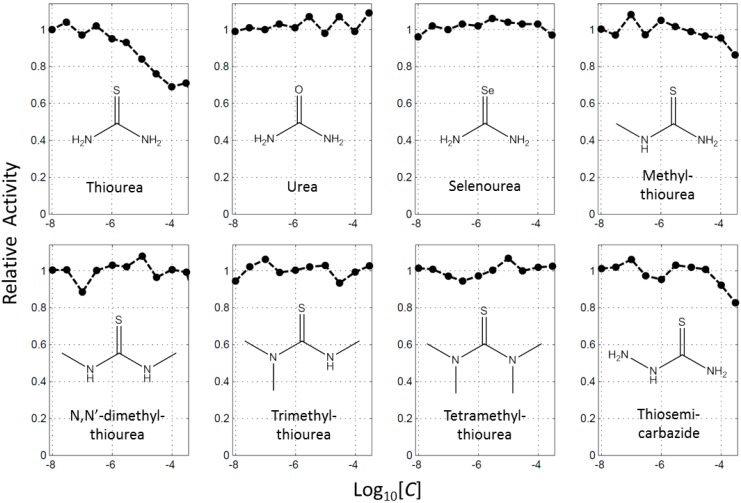
Profiles of concentration dependent inhibition of tyrosinase by thiourea and its analogues. Each inhibitory activity was scaled with that in the absence of inhibitor as 1.

BindingDB (2015m6 version) included 469 small molecules that directly inhibit tyrosinase. Of them, 139 molecules had more than a sulfur atom, and 107 contained thione. Here, the number of thiourea-containing compounds was 78, about 17% (78/469) of total tyrosinase inhibitors (Figure S1). The representative molecule of thiourea-containing inhibitors is PTU. More than 100 papers have described the effects of PTU on tyrosinase, melanoma, melanocyte, and pigmentation since the 1940s [19,20,21]. The high content of the thiourea moiety in the inhibitors raised the question whether thiourea itself would inhibit the function of tyrosinase. We observed that thiourea decreased tyrosinase enzyme activity in a concentration dependent manner (Figure 1). This result is consistent with what DuBois and Erway reported about 70 years ago [21]. On the other hand, urea or selenourea had no effect on activity, implying that the sulfur atom is important for the inhibition of tyrosinase by thiourea (Figure 1). We further investigated the roles of the nitrogen in thiourea using its analogues. Enzyme assays with mono-, di-, tri-, and tetramethylthiourea, and thiosemicarbazide showed that the effects by the molecules were much weaker than thiourea (Figure 1). The molecules of di-, tri-, and tetra-methylthiourea hardly inhibited tyrosinase at 300 μM. The data indicate that the inhibitory activity of thiourea originated from both the sulfur and the nitrogen atoms.

### 2.2. Thiourea-Containing Drugs Exhibit Inhibition of Tyrosinase

**Figure 2:**
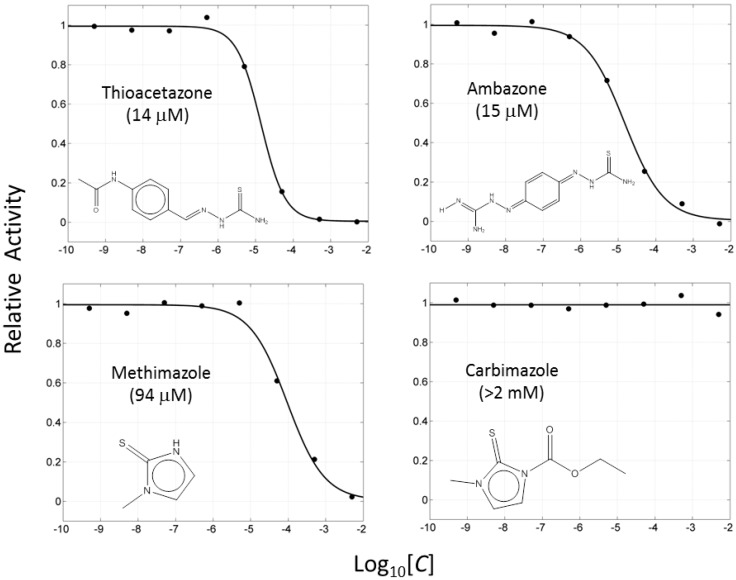
Concentration-dependent inhibitory activities of the representative compounds. Activity is normalized to come under the values in the range of 0 to 1. IC_50_ values are written in parentheses.

If this finding is true, it follows that other molecules containing thiourea may inhibit tyrosinase as well. We searched for the molecules from ZINC subsets that are in clinical use as drugs and contain a thiourea moiety [22,23]. Nine thiourea-containing drugs were retrieved, including thioacetazone, ambazone, methimazole, carbimazole, thiourasil, methylthiourasil, propylthiourasil, timiperone, and albutoin. Based on ease of acquisition, we selected seven molecules for further assessment, excluding timiperone and albutoin. Thioacetazone (also called as thiacetazone) is an anti-tuberculosis drug [24]. Ambazone is an oral antiseptic used in Europe and has garnered attention as an antineoplastic drug [25]. The other five molecules (methimazole, carbimazole, thiourasil, methylthiourasil, and propylthiourasil) are antithyroid drugs [26]. Of them, carbimazole is a prodrug that is converted to methimazole after absorption. Extensive survey of publications revealed that there was no data on the tyrosinase-related activity of the molecules, except for methimazole. Several reports are available that described the effect of methimazole on tyrosinase at the enzymatic and cellular levels [27,28,29,30]. Remarkably, we found that six of the seven molecules (the exception was carbimazole) inhibited mushroom tyrosinase. In ascending order, the half-maximal inhibitory concentration (IC_50_) values of thioacetazone, ambazone, methimazole, thiourasil, methylthiourasil, propylthioruasil, and carbimazole were 14, 15, 94, 215, 266, 375 μM, and >2 mM, respectively (Table 1, and Figure 2 and Figure S2). All were weaker than PTU (1 μM), but thioacetazone and amabazone were two-fold stronger than another well-known inhibitor, kojic acid (29 μM). Please note the similarity of the IC_50_ of PTU in this study and the reported value, 1.8 μM [31].

**Table 1:** Details of the compounds tested in this study for the inhibition of mushroom tyrosinase.

ID ^†^	Chemical	2D Structure	ZINC ID	*M*w	IC_50_ (μM)	LE ^‡^
1	Phenylthiourea (PTU)	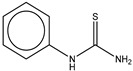	ZINC03875720	152	1	0.84
2	Kojic acid	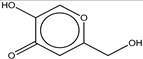	ZINC13831818	142	29	0.64
3	Thioacetazone	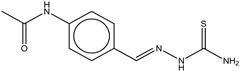	ZINC17970372	236	14	0.43
4	Ambazone	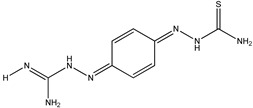	ZINC18066619	237	15	0.42
5	Methimazole	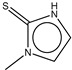	ZINC01187543	114	94	0.81
6	Carbimazole	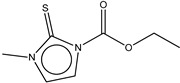	ZINC00001091	186	>2000	-
7	Thiouracil	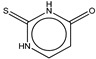	ZINC05127810	128	215	0.64
8	Methylthiouracil	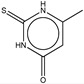	ZINC05037820	142	266	0.56
9	Propylthiouracil	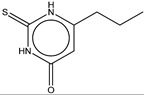	ZINC04640636	170	375	0.44

^†^ All chemicals in following Table 2, Table 3 and Table 4 have the identical IDs to those in Table 1; MW and IC_50_ mean molecular weight and half maximal inhibitory concentration, respectively; ^‡^ LE indicates ligand efficiency that is defined as 1.4 × (−log_10_IC_50_)/*N*, where *N* is the number of non-hydrogen atoms [32].

### 2.3. Thiourea-Containing Drugs Exhibit Non-Competitive Inhibitory Kinetics

**Figure 3:**
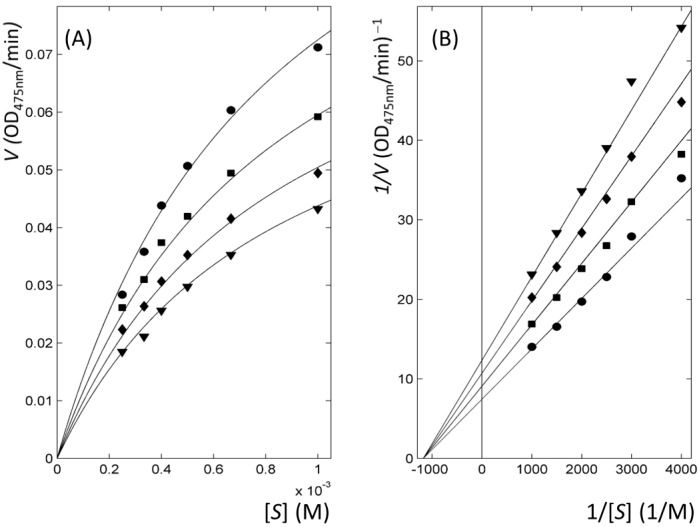
Representative kinetics of enzyme inhibition with thioacetazone. (**A**) Michaelis–Menten; and (**B**) Lineweaver–Burk plots. The lines with ▼, ♦, ■ and ● symbols correspond to the profiles at the inhibitor concentrations of 5, 10, 15, and 20 μM, respectively.

The kinetics of enzyme inhibition classified all thiourea-containing drugs as non-competitive inhibitors, whereas the reference molecules (PTU and kojic acid) were assigned as competitive inhibitors. *F*-statistics via the comparison of nonlinearly fitted χ^2^ values in four kinetic models enabled the selection of the most appropriate model for each inhibitor and thereby the extraction of the kinetic parameters (Table 2 and Figure 3). Double-reciprocal Lineweaver–Burk plots from Michaelis-Menten profiles clearly represented the mechanism (Figure 3 and Figure S3). Our results totally agreed with the previous data that reported PTU and kojic acid as competitive inhibitors [18,33,34]. However, the classification of methimazole as non-competitive inhibitor differed from the data that assigned methimazole as mixed inhibitor [27]. In methimazole, the reduced χ^2^ values, the χ^2^ values divided by the degree of freedom for *F*-statistics, were 0.32 and 0.28 for non-competitive and mixed models, respectively. It indicates that the goodness of fitting is apparently indistinguishable in two models (Figure S4). Nevertheless, our statistical analysis defined methimazole as non-competitive inhibitor. It implies the decrement of the reduced χ^2^ value by the addition of a parameter from non-competitive to mixed mechanism is insignificant. The assigned model is reasonable considering that the other antithyroid drugs are non-competitive inhibitors. The soundness of our analyses is also reflected by qualitative agreement in the order of the values of the IC_50_ and fitted inhibitory constant, *K*_i_ (Table 1 and Table 2).

**Table 2:** The inhibitory mechanisms and constants from enzyme kinetics with inhibitors.

ID	Mechanism	*K*_i_ or *K*_ic_ (μM) ^†^
1	Competitive	0.2
2	Competitive	28
3	Non-competitive	18
4	Non-competitive	9
5	Non-competitive	73
7	Non-competitive	22
8	Non-competitive	170
9	Non-competitive	196

^†^ *K*_i_ and *K*_ic_ indicate the calculated dissociation constants of the protein-inhibitor complex by nonlinear data fitting for corresponding competitive and non-competitive models, respectively.

### 2.4. Ambazone Decreases Significantly Melanin Content in Mammalian Melanoma Cells

The 3-(4,5-dimethylthiazol-2-yl)-2,5-diphenyltetrazolium bromide (MTT) and melanin content assays were performed with mammalian melanoma B16F10 cells to examine the cytotoxicity and the cell-level activity of the compounds, respectively. Since there was no cytotoxicity up to the concentration of 20 μM, melanin content assays were conducted using the same concentration. The addition of 100 μM 3-isobutyl-1-methylxanthine (IBMX) to B16F10 cells activated tyrosinase-mediated melanogenesis, leading to an elevation in melanin content. Melanin content does not reach this level, when co-incubated with an inhibitor. Of the new thiourea-containing inhibitors, ambazone significantly decreased melanin content by 20%. The magnitude of this decrease was smaller than that with PTU (40%) but much larger than that observed with other known inhibitors of kojic acid (<5%) (Table 3). The remaining five molecules, however, did not significantly affect melanin content. Thus, the inhibitory activity detected enzymatically was different from that evaluated in cells, as often occurs in the drug discovery process. This disagreement may be due to the pharmaceutical properties of the different drugs, including cell permeability and cell-type dependent non-specific protein-ligand interactions. The structural difference of target proteins, those used for enzyme activity and cellular activity, could result in this inconsistency as well. Notably, the sequence similarity of the two proteins, mushroom tyrosinase and mammalian tyrosinase, is around 30%.

### 2.5. Thiourea-Containing Drugs Also Inhibit Mammalian Tyrosinase

Enzyme assays with cell extracts of B16F10 cells were performed to confirm whether the inhibitors also affect mammalian tyrosinase. Despite the lower concentration of tyrosinase in the lysates, the absorbance changed due to the production of chromogenic products. The results were qualitatively consistent with those obtained in the enzyme-based assay with mushroom tyrosinase (Table 1 and Table 3). Five molecules, thioacetazone, ambazone, thiourasil, methylthiourasil, and propylthiourasil, significantly inhibited the function of tyrosinase, whereas the effect of methimazole was limited. There was, however, little correlation between the enzyme activity assay and the melanin content assay. In addition to the pharmaceutical and structural differences mentioned in the previous section, polypharmacology may bring about these discrepancies between the two assays [35]. Binding to off-targets in a cell type dependent manner can alter the effective concentration of a chemical, as reflected by the variation of efficacy of anticancer drugs in several cells. We explored the known cellular targets of the inhibitory molecules using ChEMBL [36,37] and BindingDB [38] databases. Several records with functional cellular assays for ambazone (CHEMBL2103762 for ChEMBL and 53352 for BindingDB) were identified. However, there was no report on the direct binding with target protein. There is some data confirmed by cell-based assay for thioacetazone (CHEMBL375492). To the best of our knowledge, the results in this study are the first to report on the activities of ambazone and thioacetazone at the enzyme level. Several papers, however, have reported the quantified activity of methimazole (CHEMBL1515) [27,28,29,30] on the several targets, including tyrosinase. The reported IC_50_ values for tyrosinase were in the range of 40 μM–1.4 mM. The value in our study, 90 μM, is within that range. The direct binding of methimazole to dopamine β-hydroxylase [39] and lactoperoxidase [40] has been reported. Although there is no report on the direct target, there are records for the cellular activities of thiourasil (CHEMBL345768), methylthiourasil (CHEMBL1330588), and propylthiourasil (CHEMBL1518). Interestingly, there is a report on the anti-thyroid effect of PTU [41], suggesting the possibility that ambazone and thioacetazone may share similar effects, although it needs to be studied further.

**Table 3:** Effects of tyrosinase inhibitors on cell survival, melanin content, and cell lysates of mammalian B16F10 cells ^†^.

ID	MTT (%)	Melanin Content (%) ^‡^	Cell Lysate (%) ^§^
	20 µM	20 µM	
1	113 ± 10	59 ± 7	5 ± 1
2	114 ± 10	108 ± 11	86 ± 8
3	95 ± 15	102 ± 10	74 ± 7
4	95 ± 12	80 ± 11	87 ± 9
5	100 ± 9	104 ± 12	93 ± 10
7	99 ± 10	106 ± 14	79 ± 9
8	108 ± 10	102 ± 14	78 ± 10
9	102 ± 12	102 ± 10	77 ± 9

^†^ Each value is the mean ± standard deviation from triplicate trials; ^‡^ Melanin content with each inhibitor was scaled with that in the absence of inhibitor (100%) after treating IBMX 100 μM to B16F10 cells; ^§^ Inhibition of the enzyme with B16F10 cell lysates was scaled with that in the absence of inhibitor (100%).

### 2.6. Docking Simulations Suggest that Thiourea Moieties of New Inhibitors Are Critical in Binding to Tyrosinase

**Figure 4:**
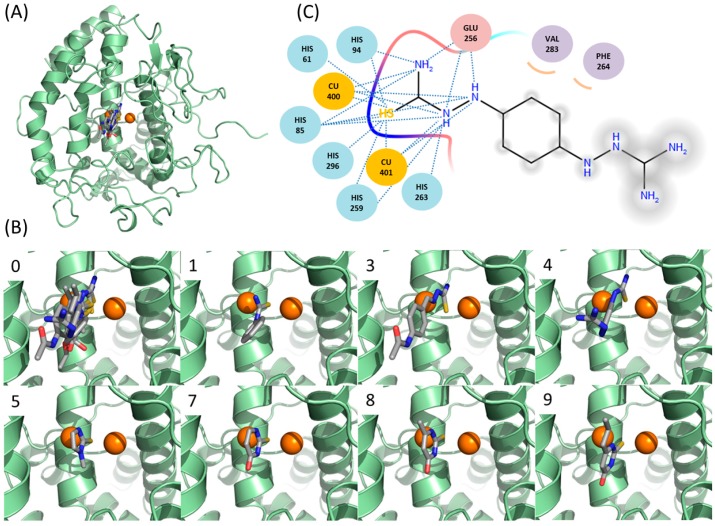
Predicted binding modes of the thiourea-containing compounds. (**A**) Overlaid inhibitors against mushroom tyrosinase. Chain A of mushroom tyrosinase [15] (PDB: 2Y9X) are used for the docking with DOCK 3.6 [42]. The sphere and grids of the protein were prepared assuming the met-state of the copper atoms with the protocol by DOCK blaster [43]. The two copper atoms in tyrosinase are drawn as orange circles; (**B**) predicted binding mode in each inhibitory compound. (0) Overlay of the inhibitors in this study, (1) phenylthiourea, (3) thioacetazone, (4) ambazone, (5) methimazole, (7) thiourasil, (8) methylthiourasil, and (9) propylthiourasil. The yellow, red, and blue colors indicate the sulfur, oxygen, and nitrogen atoms, respectively. All the figures are prepared by Pymol [44] and arranged in the same orientation; and (**C**) schematic diagram of the representative interaction between ambazone and tyrosinase. Residues in tyrosinase are drawn in circles. Residues for hydrophilic and hydrophobic interactions are coloured in pink and purple, respectively. Dashed lines correspond to hydrophilic interactions. Curves indicate hydrophobic contacts. Atoms exposed to solvent exposure are shaded.

In the complex structure, the thiourea moiety of PTU lies at the position bridging two coppers between PTU and catechol oxidase [14]. Although the limited sequence identity (<20%) between catechol oxidase and mushroom tyrosinase makes the direct translation of the binding mode of PTU into mushroom tyrosinase opaque, it is obvious that the sulfur should occupy the position. In this study, we performed docking simulation with DOCK 3.6 to understand the binding modes of the molecules at the atomic level. The reliability of DOCK 3.6 was assured in the dockings between PTU and tropolone, and mushroom tyrosinase (PDB ID: 2Y9X) [15]. The docked pose of PTU and tropolone were fairly similar to those found in the X-ray structures (Figure 4 and Figure S5). Docking simulations of the molecules found that all the sulfur atoms were located at the same position to that of PTU (Figure 4 and Figure S5), supporting the reliability of the results. The models satisfactorily explain why the thiourea unit is necessary for binding to tyrosinase. In the case of ambazone, the thiourea moiety makes extensive hydrophilic contacts with the catalytic core surrounding di-copper ions of tyrosinase. Hydrogen bond between the nitrogens of the inhibitor and the side-chain of Glu-256 is also involved in the interaction. Hydrophobic contacts with two residues, Phe-264 and Val-283, assist the interaction. Comparison with methimazole may explain why carbimazole hardly binds to tyrosinase. Different from methimazole, carbimazole cannot form an intermolecular hydrogen bond with Glu-256, because an ethoxycarbonyl group occupies the nitrogen that methimazole uses for the hydrogen bond. The data are in agreement with the enzyme assays with mono-, di-, tri-, and tetramethylthiourea (Figure 1), where any addition of methyl to the nitrogen of thiourea impaired the effect observed in thiourea. It should be stressed that all thiourea-containing inhibitory molecules in the database have free nitrogen that can act as a hydrogen bond donor (Figure S1). The intermolecular energies predicted by DOCK 3.6 were −59.1, −51.4, −43.9, −37.8, −37.2, and −35.0 (kcal/mol) for ambazone, thioacetazone, methimazole, methylthiourasil, thiourasil, and propylthiourasil, respectively. Remarkably, there was a strong correlation between IC_50_ values and the docking energies. The logarithmically scaled IC_50_ values (logIC_50_) and the energies in six thiourea-containing drugs revealed the Pearson correlation coefficient (*R*) of 0.960 (Figure S6). It also reflects the reliable performance of the docking simulation with DOCK 3.6. The moieties of thiosemicarbazone in ambazone and thioacetazone have additional nitrogen that is involved in the intermolecular hydrophilic contacts. However, the additional nitrogen is unlikely related with the stronger inhibitions, since ethionamide that contains thioamide, but not thiourea, potently inhibited tyrosinase with IC_50_ of 4 μM [18]. Rather, other intermolecular interactions including hydrophobic contacts with Phe-264 and Val-283 seem to contribute more to the strong inhibition of ambazone and thioacetazone. Future studies using biophysical methods, such as X-ray crystallography and molecular dynamics simulation, may provide the detailed explanation of the interaction between inhibitors and tyrosinase extending our clues.

### 2.7. Cheminformatics Identifies Antithyroid Drugs as a New Type of Inhibitors

**Figure 5:**
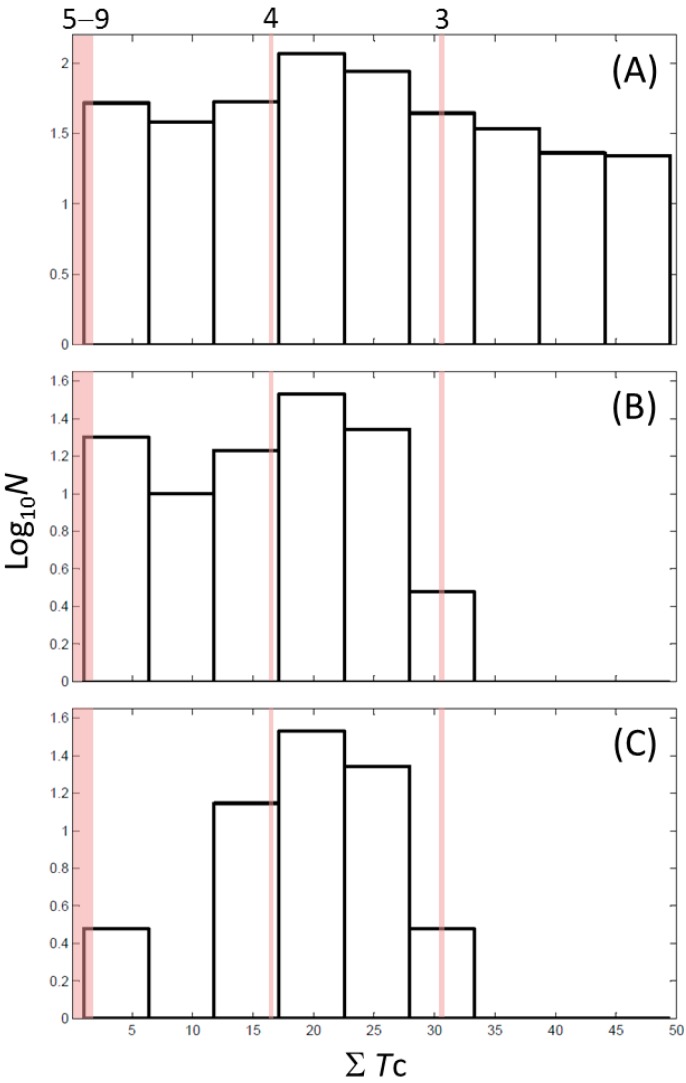
Chemical similarity to known tyrosinase inhibitors. Distribution of Σ*T*c values in (**A**) all 469 inhibitors; (**B**) 107 thione-containing inhibitors; and (**C**) 78 thiourea-containing inhibitors from BindingDB [38]. The values of Σ*T*c were calculated between all the inhibitors and individual sets of inhibitors. (**A**–**C**) have the same bin ranges in *X*-axis. The counts (*N*) in *Y*-axis are scaled logarithmically for clarity. The shaded ranges with pink colour in (**A**–**C**) corresponds to 3, 4, and 5–9 compounds with the values of Σ*T*c described in Table 4.

Next, we compared the new inhibitors in this study and known tyrosinase inhibitors by using the similarity ensemble approach (SEA) [45]. The SEA integrates the Tanimoto coefficient (*T*c) values between a test molecule and the known inhibitors (Σ*T*c) for a set of known inhibitors in a protein. The larger the Σ*T*c value, the more similarities the molecules share with the known inhibitors, whereas a smaller value implies that the molecule is an inhibitor with a distinct topology. The SEA originally used the ECFP4 fingerprint for calculating *T*c, but we employed Morgan circular fingerprint implemented in RDKit, because RDKit is freely available and generates a similar value as ECFP4. BindingDB (2015m6 version) included 469 small molecules that inhibit tyrosinase through direct binding from all species. The distributions of the Σ*T*c values that were calculated with each inhibitor were drawn as a histogram (Figure 5A). Here, the *T*c values smaller than 0.2 were ignored for calculating Σ*T*c. The average of the Σ*T*c values from the known inhibitors was 21.9 (±11.5), and the respective values for ambazone, thioacetazone, methimazole, thiourasil, methylthiourasil, and propylthiourasil were 15.9, 30.3, 0.0, 1.4, 1.3, and 1.8 (Table 4). The values of ambazone (15.9) and thioacetazone (30.3) corresponded to 121st and 376th when compared with those 469 known tyrosinase inhibitors, respectively. Therefore, the compounds sharing similarity with ambazone and thioacetazone were not rare among inhibitors. In particular, the chemical features of thioacetazone were fairly popular among thione- and thiourea-containing inhibitors (Figure 5B,C). In contrast, those of antithyroid drugs revealed much smaller Σ*T*c values. BindingDB does not contain methimazole as a tyrosinase inhibitor, although inhibition by the molecules has been reported. The known tyrosinase inhibitors that bear the closest similarity to ambazone and thioacetazone are cyclopentanone thiosemicarbazone (ZINC17730060, *T*c = 0.41, IC_50_ = 170 nM) [46] and 4-fluorobenzaldehyde thiosemicarbazone (ZINC00102508, 0.61, 11 μM) [47], respectively (Table 4). The extent of enzyme inhibition by the molecules is stronger than those observed for ambazone and thioacetazone, whereas their cellular activity has not been evaluated. The closest known inhibitors to antithyroid drugs are ZINC00509439, ZINC45320828, and ZINC45335758 (Table 4), whose quantified inhibition were reported as 1 μM (*K*_i_) [48], 70 μM (IC_50_) [49], and 179 μM (IC_50_) [49], respectively. One may criticize about the weak inhibition of antihyroid drugs as tyrosinase inhibitor. However, it should be noted that the values of ligand efficiency (LE), a metric reflecting the potential for further chemical modification, are high compared with the known inhibitors [32]. The LE values of methimazole, thiourasil, methylthiourasil, and propylthiourasil are 0.81, 0.64, 0.56, and 0.44, corresponding to 13th, 40th, 67th, and 114th, respectively, among 454 inhibitors for which we can calculate LE (Table 1).

**Table 4:** Quantified chemical similarity to known tyrosinase inhibitors.

ID	Σ*T*c ^†^	Max *T*c ^‡^	Closest Known Inhibitor ^§^	IC_50_ (μM) ^||^
3	30.5	0.61	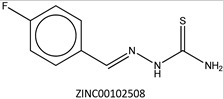	11
4	15.9	0.41	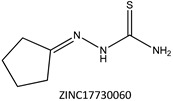	0.17
5	0.0	0.19	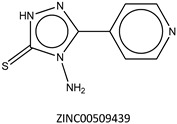	1
7	1.4	0.26	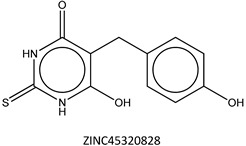	70
8	1.3	0.25		
9	1.8	0.25	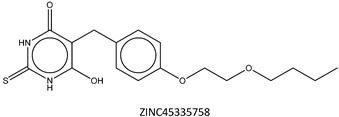	179

^†^ *T*c between the test material and 469 known tyrosinase inhibitors from the BindingDB database [38] are summed as individual values for Σ*T*c; ^‡^ “Max *T*c” in each compound indicates the closest similarity to the known inhibitors; **^§^** ZINC ID of the closest known inhibitor to each compound is written beneath 2D structure; **^||^** BindingDB-registered IC_50_ values are written for the corresponding molecules. In ZINC00509439, the value indicates *K*_i_.

## 3. Materials and Methods

Cheminformatics—The similarity between two chemicals was quantified with *T*c that used Morgan circular fingerprints implemented in RDKit (http://www.rdkit.org). Fingerprints digitized functional moieties in a molecule. *T*c evaluated the common over the union features of the digitized moieties in two molecules. *T*c has values between zero and one, where zero and one correspond to no and perfect overlap of two chemicals, respectively. Databases of ZINC [22,23,50], BindingDB [38], and ChEMBL [36,37] were employed to search for bioactive small molecules. In-house written scripts, Automated LIgand Search for PolyPharmacology (ALIS-PP), automated the cheminformatics procedure.

Enzyme activity and kinetics experiments with inhibitors—All chemicals were purchased from Sigma-Aldrich (St. Louis, MO, USA) or Tokyo Chemical Industry (Tokyo, Japan). The reaction mixture for the enzymatic assay was comprised of mushroom tyrosinase, 675 μM l-tyrosine as substrate, and inhibitors in phosphate-buffered saline. The solutions for measuring enzyme activity contained 5% dimethyl sulfoxide (DMSO) for solubilizing organic molecules. Due to the limited solubility of selenourea in DMSO, the inhibition by urea, thiourea, and selenourea was compared in the condition where DMSO was not included. The solution containing enzyme and inhibitor was incubated at 30 °C for 10 min. After adding substrate to the solution, the increments of absorbance at 475 nm were measured. After confirming the inhibition at a single concentration of 50 μM, the activities were quantified by calculating IC_50_ with a series of inhibitors at different concentrations. All experiments were conducted in triplicate. For the kinetics of enzyme inhibition, substrates at concentrations of 0.25, 0.33, 0.4, 0.5, 0.67, and 1 mM were chosen. The concentrations for each inhibitor were arranged to include the IC_50_ value. Four models (competitive, uncompetitive, non-competitive, and mixed) were extended from Michaelis–Menten equation for the extraction of the kinetic parameters (*V*_max_, *K*_m_, *K*_ic_, and *K*_iu_) [51]. The parameters of *V*_max_, *K*_m_, *K*_ic_, and *K*_iu_ mean the maximum velocity, Michaelis constant, and dissociation constants between the substrate-free enzyme and inhibitor, and the substrate-bound enzyme and inhibitor, respectively. The simultaneous nonlinear fitting with the data from all concentrations of substrate and inhibitor generated the kinetic parameters in each model with minimized χ^2^ value. The χ^2^ is defined as the sum of the squared deviations between experimental and fitted values [52]. Comparison of χ^2^ values between the four models by means of *F*-statistics enabled the selection of the most appropriate model [52,53]. All the fittings and statistical analyses in this study were performed with MATLAB^®^ (MathWorks, Natick, MA, USA).

Cell-based activity assays with inhibitors—The B16F10 murine melanoma cell in this study was purchased from the Korean Cell Line Bank (Seoul, Korea). The cells were maintained in Dulbecco’s Modified Eagles Medium (DMEM) supplemented with 10% fetal bovine serum and 1% penicillin-streptomycin under 5% CO_2_ at 37 °C. Cell viabilities were determined by 3-(4,5-dimethylthiazol-2-yl)-2,5-diphenyltetrazolium bromide (MTT) assay. In the melanin content assay, extracellular melanin release was measured. The B16F10 cells were grown until reaching the density of 1 × 10^5^ cells in 24-well plates. After adding 100 μM 3-isobutyl-1-methylxanthine (IBMX) and the inhibitors (20 μM) of this study, the cells were transferred to 96-well plates, and incubated for 48 h. Melanin content was measured using densities at 405 nm and expressed as relative percentages of untreated controls. Inhibition of tyrosinase was estimated using the cell lysates of B16F10 cells. After lysing cells with phosphate-buffered saline containing 2 mg/mL aprotinin, 10 mM leupeptin, 1 mM 4-(2-aminoethyl)-benzenesulfonyl fluoride hydrochloride (AEBSF), and 1% (*w*/*v*) Triton X-100, cells were centrifuged at 12,000× *g* for 5 min, and the supernatants were prepared as lysates for the enzyme reaction. Lysates (40 μL) were added to 100 μL DOPA (2 mg/mL) in the lysis buffer without Triton, and the absorbance change at 475 nm was observed in a time-dependent way. Inhibition was quantitated as the relative decrements of the absorbance compared with those of untreated controls.

Modeling of 3D complex structures between mushroom tyrosinase and inhibitors—Docking simulations were tried with DOCK 3.6 [42]. In-house written protocol, Automated pLatform for Integrative Structure-based DOCKing (ALIS-DOCK), automated all the processes. The procedures in principle followed what DOCK Blaster had previously described [43]. After adding protons into the apo structure (A chain of 2Y9X) by pdb2pqr server [54], only the protons that are bonded with nitrogen or oxygen were remained and renamed for the compatibility with DOCK 3.6. Sphere and grid generation for the area around the ligand of 2Y9X-A, tropolone, followed. The docking parameters for the degree of ligand sampling were 0.2 Å, 0.1 Å, and 1.2 Å for bin size, bin size overlap, and distance tolerance, respectively. Ligand desolvation corrected the scoring function that consists of intermolecular electrostatic and van der Waals’ interaction energies [42]. Molecules of flexibase format with ligand desolvation scoring term were adapted from the ZINC database [22,23].

## 4. Conclusions

*In silico* methods can accelerate drug repositioning. Of the protein- and ligand-centric approaches by computations, our study provides an example of a ligand-based method. Previously, we searched for Food and Drug Administration (FDA)-approved drugs that share chemical similarity to PTU and identified ethionamide and its analogues as new inhibitors of tyrosinase [18]. Our current strategy was to focus on drugs containing a widely existing functional unit. Since a thiourea moiety is shared in known inhibitors, we postulated that thiourea is a functional unit. Our results using enzyme and cell assays supported this hypothesis. Thioacetazone and ambazone exhibited higher inhibitory activities than kojic acid. Ambazone also decreased melanin content more than kojic acid. However, the existence of a thiourea moiety was not sufficient to inhibit tyrosinase, as the thiourea-containing molecule carbimazole did not inhibit tyrosinase. Experiments with analogues of thiourea demonstrated the necessity of the free nitrogen for intermolecular interaction. Our approach can be used for drug repositioning of other drugs in a straightforward way. Rapid increments in the publicly-available database for small molecules and high throughput screenings may reinforce this approach. The curated direct inhibitors for individual proteins, for example, those reported for detecting off-targets in SEA [45,55,56], can be combined. Our results in this study will be a helpful addition for the design of future research in this area.

## Supplementary Materials

Supplementary materials can be found at http://www.mdpi.com/1422-0067/16/12/26114/s1.
