# Supplementary material for: Repositioning of Thiourea-Containing Drugs as Tyrosinase Inhibitors
Source: Int J Mol Sci. 2015 Dec 2;16(12):28534–48. doi: 10.3390/ijms161226114 (PMC4691061; doi:10.3390/ijms161226114)
Supplement: Supplementary file 1 [file ijms-16-26114-s001.pdf]

# Supplementary Materials

# Repositioning of Thiourea-Containing Drugs as Tyrosinase Inhibitors

**Joonhyeok Choi and Jun-Goo Jee**

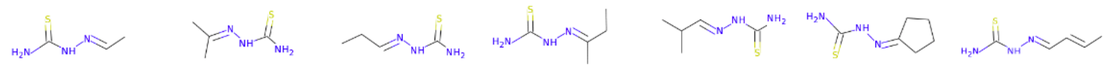

ZINC40845149 ZINC03865048 ZINC05082344 ZINC08579328 ZINC17352547 ZINC17730060 ZINC08579339

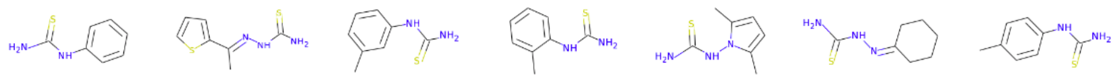

ZINC03875720 ZINC16890891 ZINC08577652 ZINC03127430 ZINC40413177 ZINC05413245 ZINC05781285

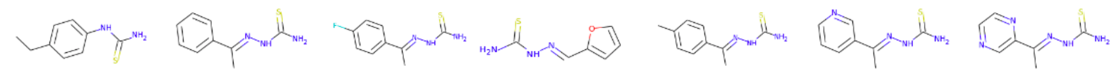

ZINC02169705 ZINC05548948 ZINC05412084 ZINC06093482 ZINC16893001 ZINC15923491 ZINC05830973

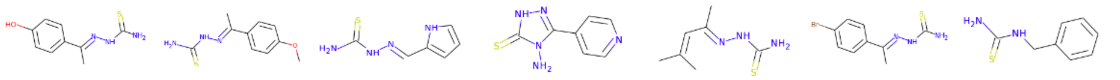

ZINC05412091 ZINC05005072 ZINC06091643 ZINC00509439 ZINC40822885 ZINC17173470 ZINC05850155

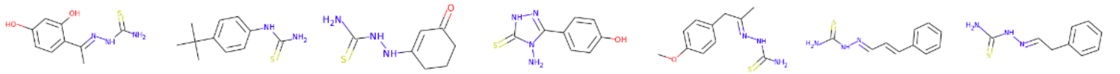

ZINC05222968 ZINC00402156 ZINC40845681 ZINC13532602 ZINC29125023 ZINC12959788 ZINC04582701

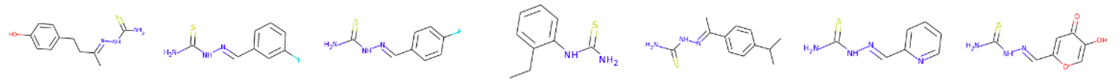

ZINC00225784 ZINC05236908 ZINC00102508 ZINC02390174 ZINC29125105 ZINC11536031 ZINC72113590

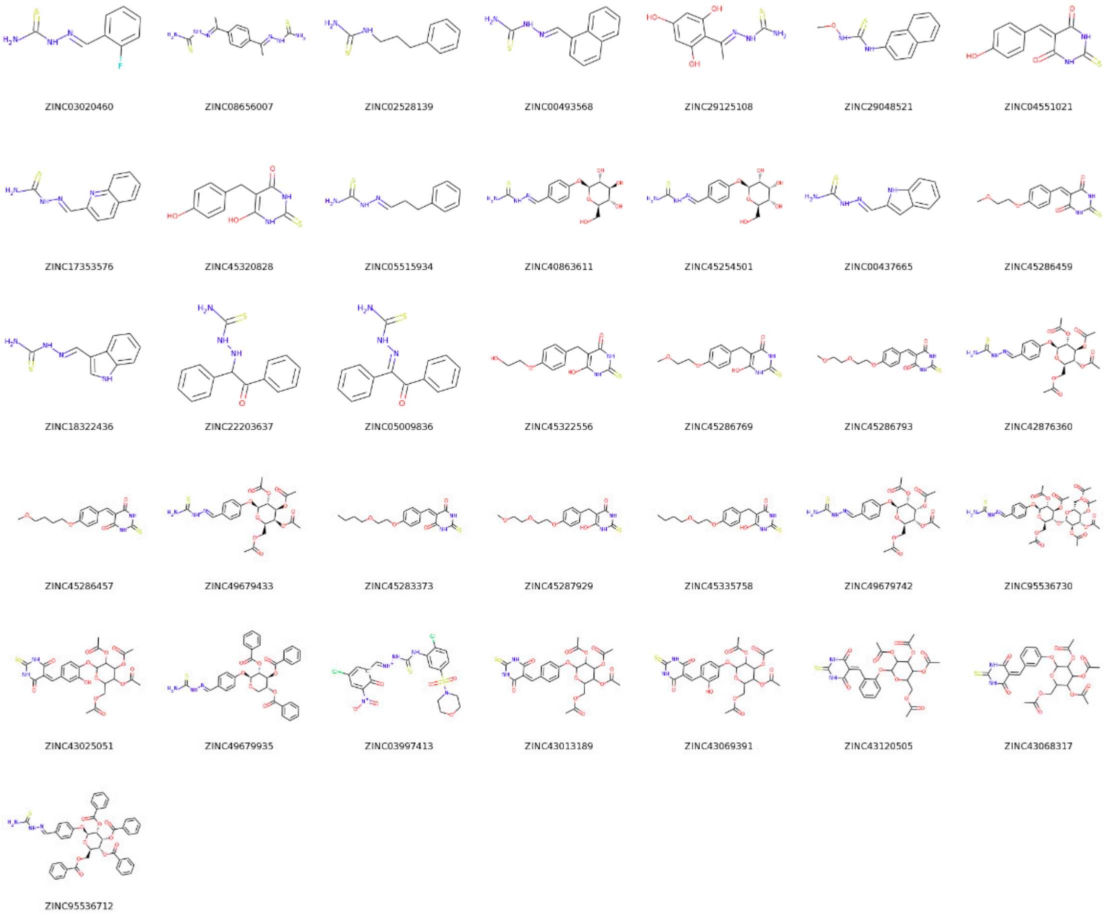


**Figure S1.** Seventy eight thiourea-containing compounds retrieved from the BindingDB database. ZINC ID of each compound is written beneath 2D structure.

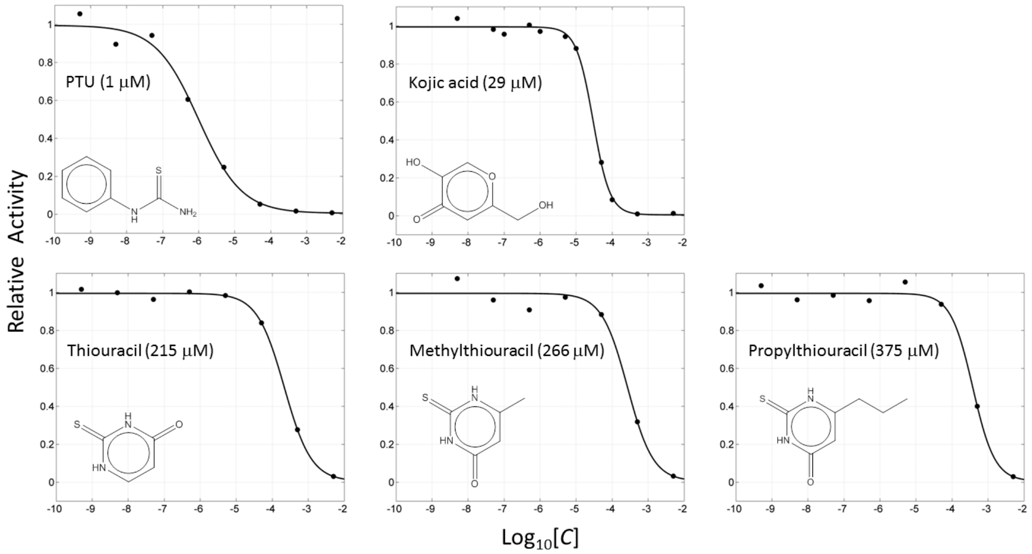


**Figure S2.** Concentration-dependent inhibitory activities of the compounds in this study. Activity is normalized to come under the values in the range of 0 to 1. The values in parentheses indicate $IC_{50}$.

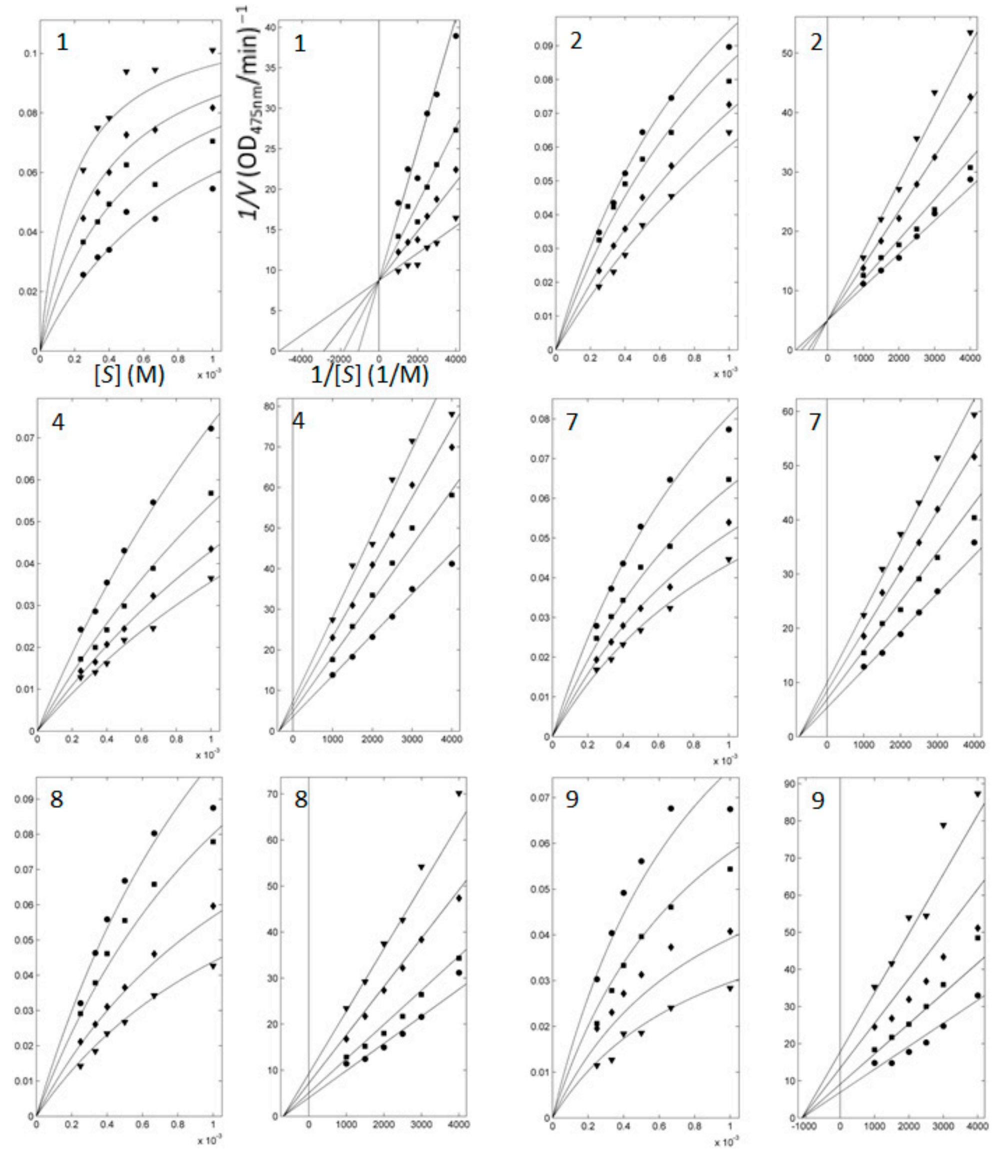


**Figure S3.** Profiles of inhibitory enzyme kinetics. (**1**) phenylthiourea (PTU); (**2**) kojic acid; (**4**) ambazone; (**7**) thiouracil; (**8**) methylthiouracil; and (**9**) propylthiouracil are drawn. Left and right figures in each compound represent Michaelis-Menten and Lineweaver-Burk plots, respectively.

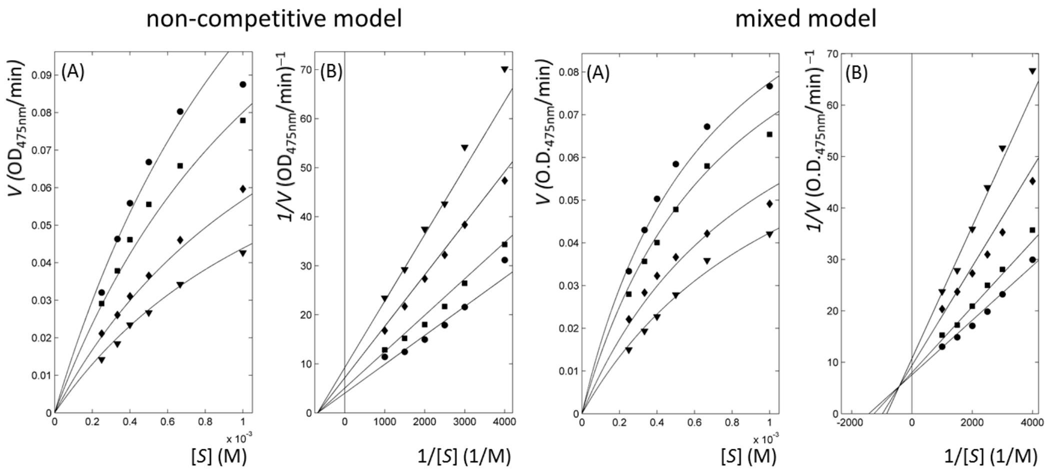


**Figure S4.** Profile of inhibitory enzyme kinetics with methimazole. The identical data were nonlinearly fitted with non-competitive and mixed models. (**A**,**B**) correspond to Michaelis-Menten and Lineweaver-Burk plots, respectively.

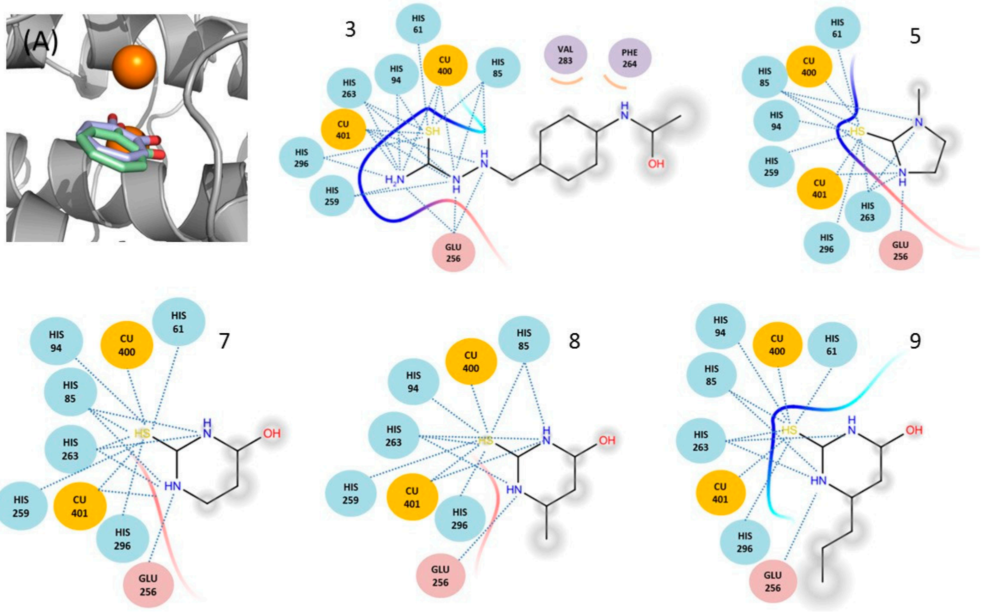


**Figure S5.** Predicted binding modes of the inhibitory compounds by docking simulation. (**A**) contains the overlaid tropolone conformations from X-ray (green) and docked (blue) structures, where orange spheres indicate copper ions. The figures of 3, 5, 7, 8, and 9 correspond to thioacetazone, methimazole, thiourasil, methylthiourasil, and propylthiourasil, respectively. Residues in tyrosinase are drawn in circle. Residues for hydrophilic and hydrophobic interactions are coloured in pink and purple, respectively. Dashed lines correspond to hydrophilic interactions, whereas curves indicate hydrophobic contacts. Atoms exposed to solvent exposure are shaded.

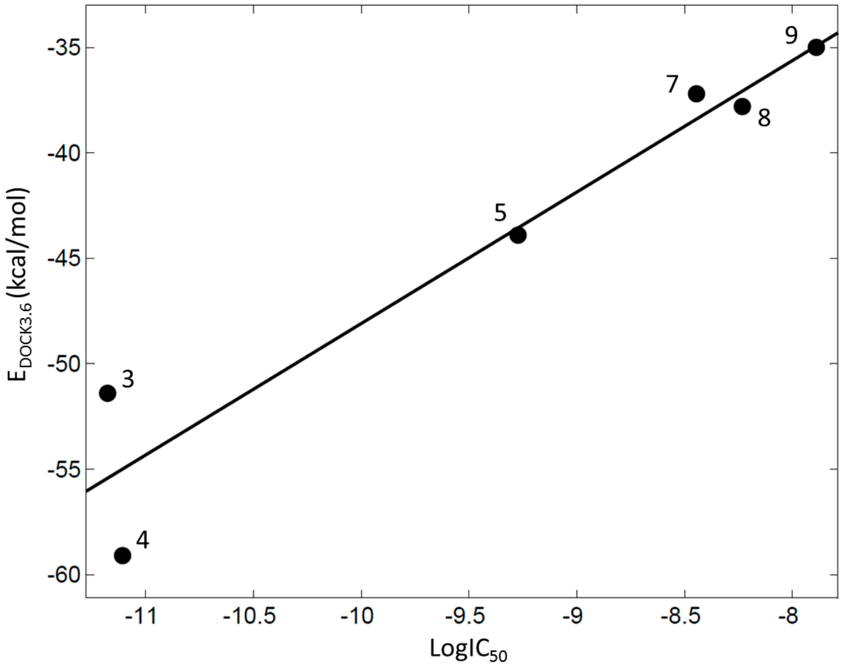


**Figure S6.** Correlation between experimental $IC_{50}$ values and simulated docking energies. The logarithmically scaled $IC_{50}$ values (*X*-axis) and the DOCK3.6-derived energies (*Y*-axis) in thiourea-containing drugs revealed the Pearson correlation coefficient (*R*) of 0.960 with $y = 6.234x + 14.241$. The points labeled with 3, 4, 5, 7, 8, and 9 correspond to thioacetazone, ambazone, methimazole, thiourasil, methylthiourasil, and propylthiourasil, respectively.
